# Spatial heterogeneity in projected leprosy trends in India

**DOI:** 10.1186/s13071-015-1124-7

**Published:** 2015-10-22

**Authors:** Cara E. Brook, Roxanne Beauclair, Olina Ngwenya, Lee Worden, Martial Ndeffo-Mbah, Thomas M. Lietman, Sudhir K. Satpathy, Alison P. Galvani, Travis C. Porco

**Affiliations:** Department of Ecology & Evolutionary Biology, Princeton University, Princeton, NJ USA; International Centre for Reproductive Health, Ghent University, Ghent, Belgium; The South African Department of Science and Technology/National Research Foundation (DST/NRF) Centre of Excellence in Epidemiological Modelling and Analysis (SACEMA), Stellenbosch University, Stellenbosch, South Africa; Francis I. Proctor Foundation, University of California, San Francisco, CA USA; Yale University, School of Public Health, New Haven, CT USA; Department of Ophthalmology, University of California, San Francisco, San Francisco, CA USA; Department of Epidemiology and Biostatistics, University of California, San Francisco, San Francisco, CA USA; School of Public Health, KIIT University, Bhubaneswar, Odisha India

**Keywords:** Leprosy, *Mycobacterium leprae*, India, Linear mixed effects regression

## Abstract

**Background:**

Leprosy is caused by infection with *Mycobacterium leprae* and is characterized by peripheral nerve damage and skin lesions. The disease is classified into paucibacillary (PB) and multibacillary (MB) leprosy. The 2012 London Declaration formulated the following targets for leprosy control: (1) global interruption of transmission or elimination by 2020, and (2) reduction of grade-2 disabilities in newly detected cases to below 1 per million population at a global level by 2020. Leprosy is treatable, but diagnosis, access to treatment and treatment adherence (all necessary to curtail transmission) represent major challenges. Globally, new case detection rates for leprosy have remained fairly stable in the past decade, with India responsible for more than half of cases reported annually.

**Methods:**

We analyzed publicly available data from the Indian Ministry of Health and Family Welfare, and fit linear mixed-effects regression models to leprosy case detection trends reported at the district level. We assessed correlation of the new district-level case detection rate for leprosy with several state-level regressors: TB incidence, BCG coverage, fraction of cases exhibiting grade 2 disability at diagnosis, fraction of cases in children, and fraction multibacillary.

**Results:**

Our analyses suggest an endemic disease in very slow decline, with substantial spatial heterogeneity at both district and state levels. Enhanced active case finding was associated with a higher case detection rate.

**Conclusions:**

Trend analysis of reported new detection rates from India does not support a thesis of rapid progress in leprosy control.

## Background

Leprosy (Hansen’s disease) is a chronic infectious disease caused by infection with *Mycobacterium leprae*, a mycobacterium closely related to the tuberculosis agent. The clinical condition of leprosy is characterized by lesions on the dermis of the skin or damage to the peripheral nerves [1, 2], and patients are classified as either paucibacillary (PB) when presenting up to five skin lesions, or multibacillary (MB) when exhibiting more than five lesions [3]. Typically, a patient demonstrates symptoms specific to either paucibacillary or multibacillary leprosy from the outset of infection, and maintains that condition throughout the duration of disease (though some borderline cases initially diagnosed as paucibacillary may later resolve into multibacillary form [4]). Patients with paucibacillary leprosy control the disease largely via cell-mediated immune pathways, while humoral immune responses are generally more pronounced among those exhibiting multibacillary characteristics [4]. Both manifestations of leprosy can be readily treated via effective multidrug therapy (MDT): a combination of rifampicin and dapsone for paucibacillary cases, with the addition of clofazimine in multibacillary cases [5]. Because of the disease’s treatability, leprosy has been a longtime target for elimination campaigns; indeed, in 1991, the World Health Assembly set a goal for “elimination of leprosy as a public health problem” by the year 2000 [5].

In spite of these goals, leprosy has proven elusive, perhaps due in part to a slow pathogen lifecycle that necessitates protracted treatment regimens [5], delays in diagnosis due to stigma (e.g. [6]), or even the possibility of asymptomatic carriers (e.g. [7]). In leprosy endemic regions, it has been suggested that most of the population will be exposed to *M. leprae* within a lifetime, though few will develop actual disease [8, 9]; indeed, approximately 5 % of the population in some endemic areas has been said to carry active *M. leprae* in nasal passageways, most without demonstrating signs [5, 10, 11]. Despite over a century of research, the mechanism of transmission for leprosy has yet to be fully resolved [2, 12]. Respiratory inhalation of aerosolized *M. leprae* particles and repeated contact with nasal mucosa and/or skin excretions are thought to play a role [13–15]. The relationship between leprosy and tuberculosis (TB, caused by *M. tuberculosis* [16]) is also of note. An inverse relationship between global and regional incidence of leprosy (decreasing) and tuberculosis (increasing) has led to the development of theories suggesting mutual exclusion between the two bacteria [17–19]. The tuberculosis vaccine, Bacille Calmette-Guérin, or BCG, infects the inoculated with an attenuated strain of *Mycobacterium bovis*, operating (with variable success) by these same principles of mycobacterium exclusion and, thus, offers some protection against leprosy [5], as well as TB (in particular, severe childhood TB [20]).

Despite these challenges, the WHO reports that the global prevalence of leprosy fell from over five million cases to fewer than 200,000 since the mid 1980s [21]. The new case detection rate, however, has remained fairly stable over the past five years. In 2012, several leading global pharmaceutical companies joined forces with the World Health Organization (WHO), the World Bank and the Bill and Melinda Gates Foundation to issue the London Declaration on Neglected Tropical Diseases. This declaration addressed several neglected tropical diseases, including two bacterial diseases—trachoma and leprosy—specifically pledging to “sustain, expand and extend programmes that ensure the necessary supply of drugs and other interventions to … help eliminate [leprosy] by 2020” [22]. The WHO currently states, “Vigorous case-finding and treatment would lead to global interruption of [leprosy] transmission by 2020, and reduce grade 2 disabilities in newly detected cases to below 1/million population at global level” [23]. Grade 2 disability is defined as visible deformity or damage present on the hands and feet, severe visual impairment, lagophthalmos, iridocyclitis, or corneal opacities [24, 25].

Most leprosy cases today are concentrated in a few nations, most particularly India, Brazil, Indonesia and the 14 other signatories of the 2013 Bangkok Declaration [26], which reaffirmed these countries’ commitment to anti-leprosy activities. Leprosy has been endemic on the Indian subcontinent since ancient times, at least as early as 2000 B.C. [27], and to this day, the region leads the world in leprosy incidence. In 2014, India accounted for more than half of the approximately 200,000 reported new leprosy cases globally [21]. Thus, India remains central to worldwide leprosy control efforts.

We used publicly available data from the National Leprosy Eradication Programme of India to explore spatial and temporal patterns in leprosy new case detection. Our aims were to: (1) estimate the rate of change in new case detections for leprosy over time and (2) estimate the extent of geographic clustering in leprosy cases to identify any district or state-level high incidence regions which may be driving nationwide trends. Additionally, we sought to investigate spatial associations in leprosy detection with (3) tuberculosis incidence, (4) BCG vaccination coverage and (5) specific clinical manifestations of disease, including the fraction of cases exhibiting grade 2 disability, the fraction occurring in children under age 15, and the fraction presented in multibacillary form.

## Methods

### Data

Spatial analysis was based on the GADM database for administrative boundaries [28] supplemented by an updated version for selected jurisdictions [29]. When districts or states were split, we combined the new districts or states into the old districts or states for consistency of reporting district over the course of the analysis.

The years, sources and spatial extent of data used in our analysis are summarized in Table 1. We analyzed district level data from the Indian Ministry of Health, which reported annual new case counts for leprosy for a (2008–2015) time series (year 2008 corresponding to the twelve month period ending March 31, 2008 and so on) [30–43]. The National Leprosy Eradication Program also provides estimated populations for each district, as well as state level estimates for the fraction of multibacillary cases, the fraction of cases among children, and the fraction with grade 2 disability at diagnosis. State-level new case data for tuberculosis, available for years 2008–2014, were obtained from reports of the Indian Ministry of Health and Family Welfare’s Revised National Tuberculosis Control Programme (RNTCP) [44]. BCG vaccination coverage estimates by state were obtained from publicly available data from the Indian government’s 2011 Evaluation Report on Integrated Child Development Services (ICDS) and were reported as averaged over a 5-year period [45]. Additionally, a group of 209 districts were identified as high endemic districts based on 2010–2011 reports [46]. These regions were targeted for subsequent enhanced activities. We entered this list of districts for use as a binary regressor. All data were extracted from PDFs using automated PDF to CSV conversion, or manual double data entry.

**Table 1:** Summary of data used in linear mixed effects regression

Data	Years available	Spatial level	Source
New case counts, leprosy	2008-2015	District	[30–43]
Enhanced case finding, 2012 and after	-	District	[46]
TB incidence	2008-2014	State	[44]
BCG coverage	Non time-varying regressor (5 yr avg)	State	[45]
Fraction exhibiting grade 2 disability	2011-2015	State	[38–42]
Fraction in children <15 years	2011-2015	State	[38–42]
Fraction in multibacillary form	2011-2015	State	[38–42]

All data utilized in linear fixed effects regression models (Tables 2 and 3), including years available, spatial extent and sources

### Statistical analysis

The primary outcome variable was the leprosy annual new case detection rate (ANCDR), defined as the number of new cases in a district divided by the estimated population of the district during that year, as published by the Indian National Leprosy Eradication Program. We conducted four analyses: spatial autocorrelation, trend analysis by linear mixed effects regression, correlations between the annual new case detection rate and other variables at the state level, and a regression analysis of the multibacillary to paucibacillary fraction. All analysis was conducted in R v. 3.1 for MacIntosh (R Foundation for Statistical Computing, Vienna, Austria), using packages sp, maptools, spdep, sperrorest and lme4.

Spatial autocorrelation was assessed by computation of Moran’s I based on the adjacency matrix. We computed the spatial correlogram out to 10 lags (connections between regions). In addition, the spatial block bootstrap (R package sperrorest) was used in assessing the significance of all regression coefficients for linear mixed effect regressions. Confidence intervals were computed using the basic bootstrap method [47].

At the district level, we fit linear mixed-effects regression models for longitudinal analysis of the annual new case detection rate [48]. Estimates were obtained for several models, each with different fixed effect predictors, but time (years) was used as a fixed effect in all. We also included the following time-varying predictors, based on the state containing the district: tuberculosis incidence, the fraction of cases exhibiting grade 2 disability, the fraction of diagnoses in children, and the fraction of cases in multibacillary form. BCG coverage was reported by state as a non-time varying regressor (a 5-year average). Finally, we used a binary indicator variable for whether or not an observed value for the annual new case detection rate in a district occurred in one of the 209 districts targeted for enhanced leprosy case detection activity after 2011 [46]. Each model included a random slope and a random intercept; an unstructured correlation matrix was assumed. We weighted the districts proportional to the population in conducting regression. To quantify the importance of predictor variables in linear mixed models, we computed the marginal and conditional *R*^2^ values [49, 50]. The marginal *R*^2^ estimates the variability explained by the fixed effect predictors; the conditional *R*^2^ estimates the variability explained by both fixed and random effects. The specific years of data used in each analysis are shown in Table 4 of the Appendix. The mathematical specification of each we examined is given in the Appendix.

We also examined the Spearman correlation between state level values of the annual new case detection rate and five predictor variables: the TB incidence rate, the extent of BCG coverage, the fraction of cases exhibiting grade 2 disability at diagnosis, the fraction of cases in children, and the fraction of leprosy cases classified as multibacillary. Confidence intervals were generated using the spatial block bootstrap at the state level.

Finally, we examined the relationship between the number of multibacillary cases and the number of paucibacillary cases in a state, using linear mixed effects regression with state as a random effect. We transformed the data according to *f*(*x*) = log(*x* + 1), and clustered by state (so that successive years from the same state were not considered independent).

## Results

### Trend analysis by linear mixed effects regression

**Fig. 1:**
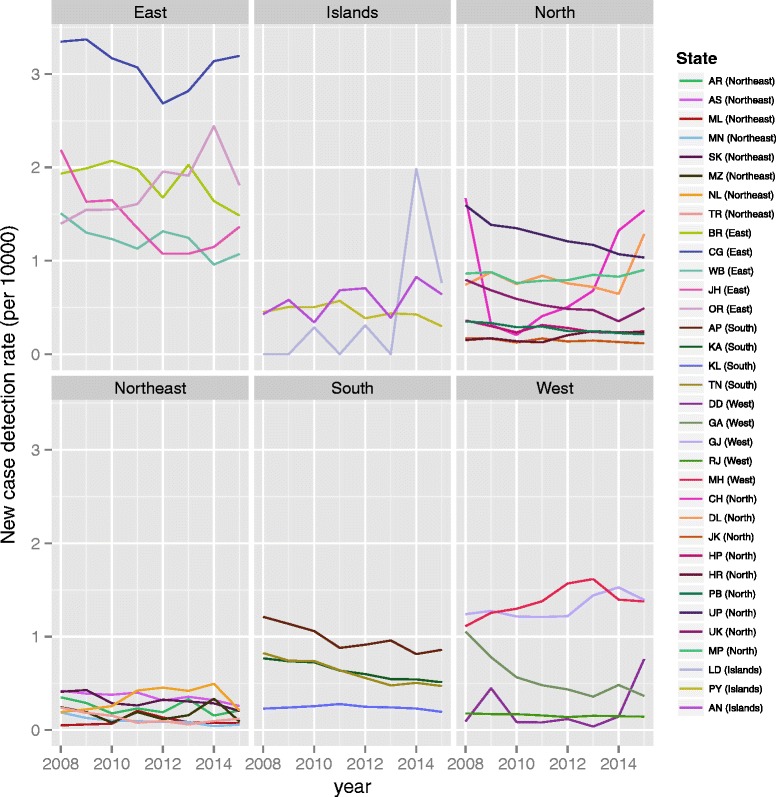
Temporal trends in new case detection rate per 10,000, by state or union territory (excluding the Union Territory of Dadra and Nagar Haveli (pop. approximately 350,000), which shows new case detection rates ranging from 3.8 to 9.9 per 10,000)

A total of 604 analytic districts in India were examined over the 2008–2015 time series. Figure 1 compiles district-level data to summarize the annual new case detection rate for leprosy by state (grouped by region), excluding the Union Territory of Dadra and Nagar Haveli (which has a small population, and a high annual new case detection rate). Pronounced differences are evident between different regions, with the Northeast showing the lowest rates.

**Fig. 2:**
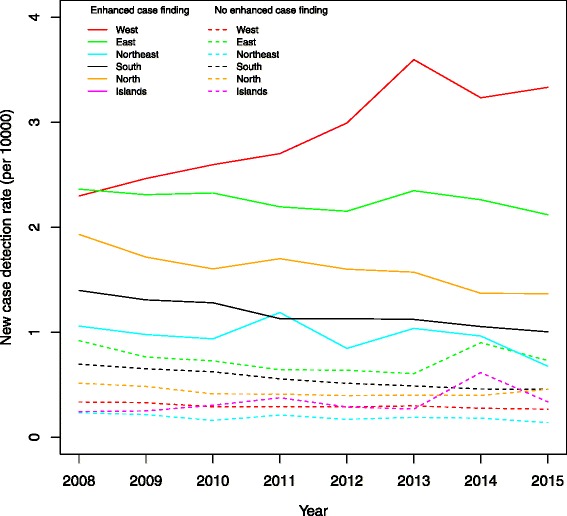
New case detection rate, by region and case finding effort. The average of districts selected for enhanced case finding activity are shown in solid; the average of other districts in dashed lines

Figure 2 shows the mean district level new case finding rate per 10,000 individuals per year, for different regions, broken down within each region into districts selected for enhanced activity and districts not selected for enhanced activity. The overall magnitude of such changes and their relation to ongoing regional trends are depicted in this figure.

We first fit a very simple linear mixed model to our district-level data including no effect of time or of any other fixed predictor, and allowed only a random intercept (representing the height of a flat trend line for each district). This model explained approximately 98 % of the variance in the annual new case detection rate (conditional *R*^2^). Using a model including only a linear time trend (not adjusting for enhanced case finding, model I in Table 2), we found the estimated overall slope (fixed effect) was − 0.0182 (with a 95 % CI of −0.026 to −0.01), *P* < 0.001. Adjusting then for enhanced case finding (model II, Table 2; including a binary indicator for every district in which enhanced case finding was conducted, if the year was 2012 or later), we found a linear trend of − 0.0237 (with a 95 % CI of −0.032 to −0.015), *P* < 0.001. Since these slopes are expressed in units of new cases per 10,000 population per year, they represent very small rates of change per year.

**Table 2:** Regression coefficients for analysis of district level new case detection rates

Model	Time trend	Covariate	Interaction	Enhanced CFA	Marginal *R* ^2^	Conditional *R* ^2^
I. Time only	− 0.0182	-	-	-	0.0014	0.993
	(− 0.027 to − 0.0035)	-	-	-		
II. Case finding activity	− 0.0237	-	-	0.0811	0.0021	0.993
	(− 0.036 to − 0.011)	-	-	(0.021 to 0.21)		
III. TB, time	− 0.0234	− 8.83 × 10^− 5^	-	0.0646	0.0016	0.994
	(− 0.04 to − 0.0058)	(− 0.00015 to 0.00047)	-	(− 0.0078 to 0.22)		
IV. TB, time, interaction	− 0.0625	− 0.00173	5.57 × 10^− 4^	0.0529	0.002	0.994
	(− 0.093 to − 0.046)	(− 0.0027 to − 0.0011)	(0.00036 to 0.00089)	(− 0.025 to 0.19)		
V. BCG, time	− 0.0236	− 0.00738	-	0.0787	0.0078	0.993
	(− 0.036 to − 0.012)	(− 0.016 to 0.0088)	-	(0.0091 to 0.21)		
VI. BCG, time, interaction	− 0.177	− 0.00924	0.00178	0.0885	0.0047	0.993
	(− 0.25 to − 0.088)	(− 0.016 to 0.0068)	(7 × 10^− 4^ to 0.0026)	(0.023 to 0.22)		
VII. Fraction grade 2, time	− 0.0171	2.34 × 10^− 4^	-	0.213	0.0073	0.993
	(− 0.03 to − 0.0062)	(0.00019 to 0.00039)	-	(0.14 to 0.39)		
VIII. Fraction grade 2, time, interaction	− 0.0195	− 9.82 × 10^− 4^	4.04 × 10^− 4^	0.213	0.0072	0.993
	(− 0.034 to 0.0043)	(− 0.0046 to 0.011)	(− 0.0034 to 0.0016)	(0.16 to 0.38)		
IX. Fraction children, time	− 0.0227	0.00379	-	0.135	0.0035	0.993
	(− 0.039 to − 0.012)	(− 0.0072 to 0.009)	-	(0.058 to 0.28)		
X. Fraction children, time, interaction	0.0166	0.0259	− 0.00496	0.144	0.0039	0.993
	(− 0.0083 to 0.041)	(0.0081 to 0.04)	(− 0.0077 to − 0.0026)	(0.081 to 0.29)		
XI. Fraction MB, time	− 0.0226	0.0567	-	0.127	0.0026	0.993
	(− 0.039 to − 0.013)	(− 0.21 to 0.16)	-	(0.046 to 0.29)		
XII. Fraction MB, time, interaction	− 0.127	− 0.83	0.178	0.142	0.0037	0.993
	(− 0.2 to − 0.083)	(− 1.5 to − 0.52)	(0.092 to 0.30)	(0.076 to 0.32)		

The specific statistical models are specified in the Appendix. All models include calendar time in years, and all models except for the base model include a term “Enhanced CFA” indicating whether a particular district-year corresponds to one of the 209 districts selected for enhanced case finding activity. Models include tuberculosis incidence (state level), the average BCG coverage (state level), percent of grade 2 disability at diagnosis (state level), percent of cases in children (state level), or the fraction multibacillary (state level). Interaction with time is omitted, and then included, in each model in turn. Marginal *R* ^2^ values indicate the fraction of variance explained by the fixed effects, and conditional *R* ^2^ indicate the fraction of variance explained by both fixed and random effects; see text. Confidence intervals derived by spatial block bootstrap (with a radius of 1.5°; see text for details)

Table 2 shows the results of additional linear mixed effects regression models of the district-level annual new case detection rates. Adding time (a fixed effect overall trend, and a random effect allowing each district to have a different rate) to the models does not (and cannot) substantially increase the overall *R*^2^, which is already very high. All models showed a gentle, but statistically significant, linear trend towards declining new case detection rates for leprosy. The linear trend was more pronounced in models considering each variable’s interaction with time. Additionally (unsurprisingly), all models in which we included the indicator for enhanced activity showed a significant effect for that variable. When increased surveillance efforts were employed in an attempt to locate more cases in specific districts, more cases were found.

**Fig. 3:**
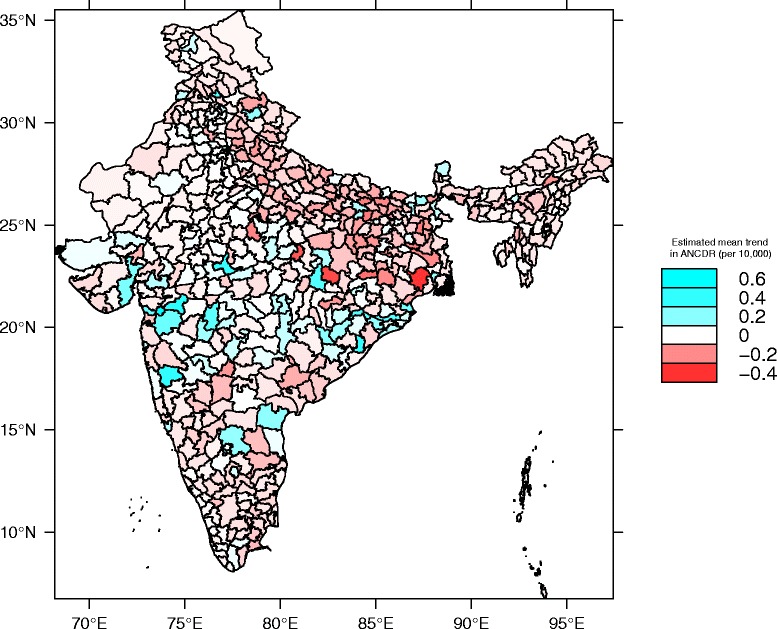
Estimated linear trend in annual new case detection rate per district, adjusting for enhanced case finding in specific districts. Red corresponds to decreasing trend; blue to increasing. Map depicts estimated district-level linear trend lines from linear mixed effects regression model (model II; see text for details)

We estimated both a fixed effect and random effect slope. Adding the estimated random effect for each district to the overall fixed effects yields an overall slope for that region. We mapped these slopes these in Fig. 3, which shows the estimated trend map by each district, adjusting for enhanced case finding. We first applied the transformation log((NEWCASES + 0.5)/POPULATION). These trends were derived from model II (Table 2), which includes an overall rate of change (linear trend), a term for enhanced case finding, and a random slope and intercept for each district. The map demonstrates the extent of spatial clustering in new case detection for leprosy across India and visibly highlights those districts with modestly large or small estimated slopes. East Midnapore district in the state of West Bengal stands out in the east (shown in red) as having the largest estimated decline, largely due to a reported over tenfold drop in new case detection in 2014 and 2015.

### Spatial autocorrelation

As a measure of spatial autocorrelation, we computed Moran’s I based on the average new case detection rate over the period 2008–2015, at the district level. The overall value was 0.124 (*P* < 0.001). Year by year, the results are similar, ranging from 0.0987 to 0.134. Examination of a spatial correlogram (up to lag 10) based on the adjacency matrix of the districts indicates that Moran’s I drops off rapidly with increasing lag, falling below 0.05 after lag 3 in nearly all cases. The median of the mean distance between the centroid of each district and those of its neighbors was approximately 74.3 km; our choice of 1.5° for spatial block bootstrap is meant to be roughly double this value. However, this analysis does not consider state boundaries or the possibility of heterogeneity in spatial scale.

### Correlations between case detection trends and state-level predictors

While the longitudinal analysis shows that the intercept (initial value for the annual new case detection rate) of each district is the overwhelmingly most important single statistical predictor of future values, it does not relate these initial values to the other predictors of interest: TB incidence, BCG coverage, fraction of cases exhibiting grade 2 disability, fraction of cases in children, and fraction of cases in multibacillary form. Table 3 shows the correlation coefficients between these quantities at the state level. No statistically significant interaction was observed between the annual new case detection rate for leprosy and TB incidence or BCG coverage at the state level. A significant positive relationship was identified between the annual new case detection rate and the fraction of cases in children, indicating that a higher proportion of cases in children <15 years correlates with higher annual case detection overall. Table 3 also shows a significant negative relationship between the annual new case detection rate and the fraction of multibacillary cases, and between the fraction in children and the fraction multibacillary. Annual case detection rates are higher when there are proportionally more multibacillary versus paucibacillary cases, and as the fraction of cases in children increases, so too does the fraction presenting in multibacillary form.

**Table 3:** Spearman correlation between leprosy annual new case detection rate and selected state level quantities

	Leprosy	TB	BCG	Grade 2	Childhood	Fraction
	ANCDR	incidence	rate	fraction	fraction	MB
Leprosy	1.0	−0.029	−0.093	−0.032	0.47*	−0.40*
ANCDR	-	(−0.48 to 0.061)	(−0.31 to 0.11)	(−0.18 to 0.15)	(0.28 to 0.70)	(−0.50 to −0.27)
TB	-	1.0	0.019	−0.11	−0.065	−0.012
incidence			(−0.21 to 0.32)	(−0.23 to −0.044)	(−0.32 to 0.18)	(−0.086 to 0.20)
BCG	-	-	1.0	−0.12	0.090	−0.008
rate				(−0.40 to −0.23)	(−0.18 to 0.28)	(−0.11 to 0.067)
Grade 2	-	-	-	1.0	0.041	−0.20*
Fraction					(−0.13 to 0.13)	(−0.11 to −0.26)
Childhood	-	-	-	-	1.0	−0.30*
Fraction						(−0.49 to −0.86)
Fraction MB	-	-	-	-	-	1.0

*statistically significant, alpha = 0.05, no adjustment for multiple comparisons

### Regression analysis of multibacillary to paucibacillary

In addition to our analysis of state-level drivers of temporal trends in leprosy case reporting, we conducted a regression of the number of multibacillary versus paucibacillary cases, using linear mixed effects regression, longitudinally analyzing the data by state. The linear term was 0.382, with a standard error of 0.109. The quadratic term is significant (*P* < 0.001); the sign (not shown) indicates that jurisdictions with fewer total cases are somewhat more likely to have a higher ratio of multibacillary to paucibacillary cases compared to those with higher case detection rates. However, we note that this effect is small. Measuring the explanatory capability of the model by the conditional *R*^2^ from regressing the data on the predicted values [28], the quadratic term only increases the conditional *R*^2^ by a very modest 0.021.

## Discussion

National, state and district level leprosy trends in India suggest a slowly changing endemic disease in very gentle decline, with the rate of new cases over the 2008–2015 time series diminishing by less than 2 % per year at the district level. These trends echo global patterns of slow leprosy decline over the past decade, following a dramatic decrease in new case reports in the early 2000s [51]. Some have argued that the substantial reduction in global reporting of new leprosy cases witnessed between the 1990s and 2000s may be the result of underdetection or changes in reporting [51]. In 209 Indian districts selected for enhanced case finding initiatives post-2011, significantly more new cases were found in subsequent years following the induction of heightened surveillance efforts. Additionally, our analysis demonstrates substantial spatial clustering and heterogeneity at both the district and state level for India, including identification of some states or districts with notable *increases* in new leprosy cases.

Whatever the case in past centuries [17–19] before widespread BCG vaccination and chemoprophylaxis, we found no evidence of a strong relationship between tuberculosis and leprosy at the state level in India. Additionally, we found no evidence of an association between new leprosy case detection rates and average BCG coverage. Such lack of correlation is hardly a surprise, given very high BCG coverage rates and the relative rarity of leprosy at this time. There is simply not enough variability in BCG coverage for the efficacy of BCG against leprosy [5] to become manifest. At the district level, the best predictor of future annual case detection rates is the past rate; at the state level, we can distinguish that annual new case detection rates are higher when the percentage of cases in children is higher and when the percentage of paucibacillary cases of multibacillary form is lower. Regression analysis of the rate of multibacillary to paucibacillary case detection by state further suggests that those states with higher total new case detection rates report higher levels of paucibacillary vs. multibacillary, possibly indicative of lowered detection rates for the more subtle paucibacillary clinical condition in regions of less intensive case surveillance.

## Conclusions

Enormous strides have been made by India and by other countries in fighting leprosy. Substantially fewer cases are reported today than in years past [21]. However, our analysis indicates both optimism and pessimism in consideration of the challenge of global leprosy reduction. While overall declining trends in new case detection rates for leprosy in India have been substantial, spatial patterns of leprosy persistence suggest that the reality of this public health burden is more nuanced. Clearly, new case detection rates can fall because the burden of disease is truly decreasing in the community. The new case detection rate could also fall because infected individuals are becoming diagnosed later, because less effort is spent on active case finding [6, 51–53], because a given effort expended in active case finding is becoming less effective as prevalence declines [25], or because of changes in reporting criteria (such as not reporting single lesion cases [53]). If active case finding activities in India were curtailed in the years following 2000–2005, as has been suggested in the literature [51–53], then reported new case detection rates may not reflect the true extent of leprosy disease. Many active enhanced case finding surveys conducted by Indian researchers in recent years have reported finding substantial numbers of new cases in specific locations [54–56], thus supporting these views.

A fall in the new case detection rate can, therefore, be favorable or unfavorable. Yet for the other bacterial disease targeted in the London Declaration—trachoma—evaluation is based on objective population surveys of clinical signs [57] and does not depend on health care seeking behavior (with active case finding by the program not typically conducted in any event). The two diseases could hardly differ more: trachoma—a common subclinical infection whose relatively uncommon late sequelae are of concern, and leprosy—a now uncommon disabling and disfiguring infection with insidious onset and a clinical course of years to decades. Yet the trachoma experience may contain a valuable lesson: active surveys (which have been conducted on relatively large scales for leprosy) could be included for a time as a routine components of leprosy evaluation [58] as they are for trachoma, given a suitable survey frequency and a cost-saving design.

Current recommendations include a goal of “interruption of transmission.” It is unlikely that transmission can be completely interrupted or stopped as long as a single undiagnosed active case exists in the world. Disease incidence and prevalence will eventually drop to zero if and only if we attain (and sustain) subcriticality—the condition that one case, on average, causes *fewer* than one new case, and thus never replaces itself [59, 60]. The reproduction number is defined as the expected number of cases that a given case of disease can directly cause in a fully susceptible population; thus, subcriticality corresponds to a reproduction number less than one. Reduction of the reproduction number for leprosy to well below the replacement value of one, through effective case finding and cure, should be our goal (e.g. [61]). Unfortunately, epidemiological trends suggest that, barring changes, leprosy will persist—and transmission will be maintained—in India for many years, likely beyond the stated 2020 goal of the London Declaration (see also [62, 63]). Far-reaching policy changes may be needed to accelerate the projected time course of leprosy reduction. Perhaps enhanced case holding and case finding [64], enhanced use of contact investigation and chemoprophylaxis [65], or newer technological developments will be the key to achieving a more rapid pace of decline.

## Appendix

### Statistical models

We fit specific linear mixed effects regression models [48].

Let *Y*_iτ_ be the annual new case detection rate in district *i* at year *τ*. For the *state or territory* containing district *i* at year *τ*, let *M*_iτ_ be the fraction multibacillary, *G*_iτ_ the fraction with grade 2 disability at diagnosis, and let *C*_iτ_ the fraction of diagnoses in children; these three variables are available for years 2011–2015. Let *T*_iτ_ be the tuberculosis incidence (per 100,000) for the state containing district *i* at year *τ*, available for years 2008–2014. Finally, let *B*_*i*_ be the average BCG coverage rate for the state containing district *i* . Let *E*_iτ_ be 1 if district *i* is one of the 209 districts selected for enhanced case finding and *τ* ≥ 2012 . Generically, we will let *X* denote any of *M*, *G*, *C*, or *T*.

Model I is

$$ {Y}_{\mathrm{i}\uptau}={a}_0+{a}_1\tau +{u}_{0^i}+{u}_{1^i}\tau +{\upepsilon}_{\mathrm{i}\uptau}, $$

where *a*_0_ is overall intercept, *a*_1_ the overall slope, ${u}_{0^i}$ is the random intercept for individual *i*, ${u}_{1^i}$ is the random slope for individual *i*, and ϵ_iτ_ is a normally distributed error term with mean 0 and standard deviation *σ*_*e*_ . Here, we assume normally distributed random effects with mean 0 and a general covariance matrix, so that writing ${u}^i={\left[{u}_{0^i},{u}_{1^i}\right]}^T$, we have *u*^*i*^ is distributed as bivariate normal with mean vector 0 and covariance matrix *D*.

Model II is

$$ {Y}_{\mathrm{i}\uptau}={a}_0+{a}_1\tau +{a}_2{E}_{\mathrm{i}\uptau}+{u}_{0^i}+{u}_{1^i}\tau +{\upepsilon}_{\mathrm{i}\uptau}, $$

where *a*_2_ is the effect of enhanced active case finding.

Models III, VII, IX and XI may be represented

$$ {Y}_{\mathrm{i}\uptau}={a}_0+{a}_1\tau +{a}_2{E}_{\mathrm{i}\uptau}+{a}_3{X}_{\mathrm{i}\uptau}+{u}_{0^i}+{u}_{1^i}\tau +{\upepsilon}_{\mathrm{i}\uptau}, $$

where *a*_3_ is the estimated effect for covariate *X* (which may be multibacillary fraction, fraction grade 2 disability at diagnosis, fraction in children and tuberculosis incidence). Note all these variables are at the state (or territory) level, so all districts within a state (or territory) have the same value at each time point.

Model V may be represented

$$ {Y}_{\mathrm{i}\uptau}={a}_0+{a}_1\tau +{a}_2{E}_{\mathrm{i}\uptau}+{a}_4{B}_i+{u}_{0^i}+{u}_{1^i}\tau +{\upepsilon}_{\mathrm{i}\uptau}, $$

where *a*_4_ is the estimated effect for average BCG coverage.

Models IV, VIII, X and XII may be represented

$$ {Y}_{\mathrm{i}\uptau}={a}_0+{a}_1\tau +{a}_2{E}_{\mathrm{i}\uptau}+{a}_3{X}_{\mathrm{i}\uptau}+{a}_5\tau {X}_{\mathrm{i}\uptau}+{u}_{0^i}+{u}_{1^i}\tau +{\upepsilon}_{\mathrm{i}\uptau}, $$

where *a*_5_ is a fixed effect interaction term between time and covariate *X*.

Finally, model VI may be represented

$$ {Y}_{\mathrm{i}\uptau}={a}_0+{a}_1\tau +{a}_2{E}_{\mathrm{i}\uptau}+{a}_4{B}_i+{a}_6\tau {B}_i+{u}_{0^i}+{u}_{1^i}\tau +{\upepsilon}_{\mathrm{i}\uptau}, $$

where *a*_6_ is an interaction between average BCG coverage and time.

**Table 4:** State and union territory abbreviations and regional classifications as used here. Data from the new state of Telengana are aggregated with Andhra Pradesh for consistency of comparison across multiple years

State or Territory	Abbreviation	Region
Andaman and Nicobar	AN	Islands
Andhra Pradesh	AP	South
Arunachal Pradesh	AR	Northeast
Assam	AS	Northeast
Bihar	BR	East
Chhattisgarh	CG	East
Chandigarh	CH	North
Daman and Diu	DD	West
Dadra and Nagar Haveli	DN	West
Delhi	DL	North
Goa	GA	West
Gujarat	GJ	West
Himachal Pradesh	HP	North
Haryana	HR	North
Jharkhand	JH	East
Jammu and Kashmir	JK	North
Karnataka	KA	South
Kerala	KL	South
Lakshadweep	LD	Islands
Maharashtra	MH	West
Meghalaya	ML	Northwest
Manipur	MN	Northwest
Madhya Pradesh	MP	North
Mizoram	MZ	Northwest
Nagaland	NL	Northwest
Odisha (Orissa)	OR	East
Punjab	PB	North
Puducherry	PY	Islands
Rajasthan	RJ	West
Sikkim	SK	Northeast
Tamil Nadu	TN	South
Tripura	TR	Northeast
Uttaranchal	UK	North
Uttar Pradesh	UP	North
West Bengal	WB	East
